# Effects of Single Injection of Local Anesthetic Agents on Intervertebral Disc Degeneration: *Ex Vivo* and Long-Term *In Vivo* Experimental Study

**DOI:** 10.1371/journal.pone.0109851

**Published:** 2014-10-06

**Authors:** Koji Iwasaki, Hideki Sudo, Katsuhisa Yamada, Hideaki Higashi, Takashi Ohnishi, Takeru Tsujimoto, Norimasa Iwasaki

**Affiliations:** 1 Department of Orthopaedic Surgery, Hokkaido University Graduate School of Medicine, Sapporo, Japan; 2 Department of Advanced Medicine for Spine and Spinal Cord Disorders, Hokkaido University Graduate School of Medicine, Sapporo, Japan; 3 Division of Infection and Immunity, Hokkaido University Research Center for Zoonosis Control, Sapporo, Japan; University of Michigan, United States of America

## Abstract

**Background:**

Analgesic discography (discoblock) can be used to diagnose or treat discogenic low back pain by injecting a small amount of local anesthetics. However, recent *in vitro* studies have revealed cytotoxic effects of local anesthetics on intervertebral disc (IVD) cells. Here we aimed to investigate the deteriorative effects of lidocaine and bupivacaine on rabbit IVDs using an organotypic culture model and an *in vivo* long-term follow-up model.

**Methods:**

For the organotypic culture model, rabbit IVDs were harvested and cultured for 3 or 7 days after intradiscal injection of local anesthetics (1% lidocaine or 0.5% bupivacaine). Nucleus pulposus (NP) cell death was measured using confocal microscopy. Histological and TUNEL assays were performed. For *in vivo* study, each local anesthetic was injected into rabbit lumbar IVDs under a fluoroscope. Six or 12 months after the injection, each IVD was prepared for magnetic resonance imaging (MRI) and histological analysis.

**Results:**

In the organotypic culture model, both anesthetic agents induced time-dependent NP cell death; when compared with injected saline solution, significant effects were detected within 7 days. Compared with the saline group, TUNEL-positive NP cells were significantly increased in the bupivacaine group. In the *in vivo* study, MRI analysis did not show any significant difference. Histological analysis revealed that IVD degeneration occurred to a significantly level in the saline- and local anesthetics-injected groups compared with the untreated control or puncture-only groups. However, there was no significant difference between the saline and anesthetic agents groups.

**Conclusions/Significance:**

In the *in vivo* model using healthy IVDs, there was no strong evidence to suggest that discoblock with local anesthetics has the potential of inducing IVD degeneration other than the initial mechanical damage of the pressurized injection. Further studies should be performed to investigate the deteriorative effects of the local injection of analgesic agents on degenerated IVDs.

## Introduction

Discogenic low back pain (LBP) is generally caused by progressive intervertebral disc (IVD) degeneration associated with aging or trauma [1]. Despite the lack of proven diagnostic validity, analgesic discography (discoblock) can be used to diagnose or treat discogenic LBP based on the level of pain relief caused by injecting a small amount of local anesthetics [2]. A needle is inserted into the nucleus pulposus (NP) under fluoroscopic guidance, and a local anesthetic agent is injected into the center of the painful IVD [2].

Lidocaine and bupivacaine are two of the most commonly used local anesthetics for injection therapy and diagnosis. However, negative effects have been found to be associated with their use, particularly cytotoxity [3]. Several *in vitro* studies reported dose- and time-dependent cytotoxic effects of these local anesthetics on IVD cells at clinically applied concentrations [3]–[7]. In clinical settings, despite the small number of patients without long-term follow-up, Ohtori et al. reported that accelerated IVD degeneration was not observed after a single bupivacaine injection among young patients [8]. However, a recent prospective, matched cohort clinical study showed that discography using small-needle disc puncture was associated with more extensive IVD degeneration at a 10-year follow-up [9], although this was a provocative discography approach using a non-irritating contrast agent. Thus, there is currently no consensus on the clinical effects of these procedures on IVD degeneration.

Although an increasing number of reports have described the cytotoxic effects of anesthetic agents on IVD cell *in vitro* [3]–[7] and animal studies have confirmed that needle puncture and limited pressurization caused IVD degeneration [10], [11], we are not aware of any published *ex vivo* studies or *in vivo* experimental data regarding the association of a single local injection of an anesthetic agent with IVD degeneration. Therefore, the present study aimed to investigate the deteriorative effects of lidocaine and bupivacaine in rabbit IVDs using an organotypic culture system and an *in vivo* single local injection model followed for up to 12 months.

## Materials and Methods

### Animals

Japanese white rabbits that weighed 3.2 to 3.5 kg (4–5 months of age) were used in this study. A total of 47 rabbits was used, including 15 for *ex vivo* experiments and 32 for *in vivo* experiments. All animal procedures in this study were specifically approved by the Institutional Animal Care and Use Committee of Hokkaido University.

### *Ex vivo* study

#### IVD Organ Culture Conditions and Experimental Protocol

After the intravenous administration of 10,000 IU of heparin, rabbits were euthanized, the whole spine (T12/L1–L5/6) was surgically harvested, and soft tissue was removed under sterile conditions [12], [13]. Functional spinal units consist of two vertebrae surrounding one disc. The superior end plate of the upper vertebra and the inferior end plate of the lower vertebra were removed. To ensure nutrition in a combined disc/endplate culture, it is a prerequisite to remove the vertebral bone from endplates [14]. We intradiscally injected 1% lidocaine (Astrazeneka, Luton, UK) or 0.5% bupivacaine (Astrazeneka) using a micro syringe (Hamilton, Reno, NV, USA) with a 26-gauge needle (15 µL per disc). A 0.9% saline solution (Otsuka Pharmaceutical Co., Tokyo, Japan) was used as a control. The injected dose was based on previous studies that described intradisc injection of a drug solution [15].

IVDs were then cultured in 6-well plates with 8 mL of Dulbecco’s Modified Eagle’s Medium (Sigma–Aldrich, St. Louis, MO, USA) supplemented with 10% fetal bovine serum (Nichirei Bioscience, Tokyo, Japan), 1% penicillin/streptomycin, and 1.25 µg/mL fungizone (Life Technologies, Carlsbad, CA, USA). The culture medium was changed every 2 days, and IVDs were analyzed 3 or 7 days after the injection. IVDs were divided into 5 groups:

- untreated control: IVDs without needle puncture;
- puncture-only group: IVDs with needle puncture, but without any reagent;
- saline group: IVDs with saline injection;
- lidocaine group: IVDs with lidocaine injection; and
- bupivacaine group: IVDs with bupivacaine injection.

#### Analysis of NP Cell Death

Dead and whole cells were detected with propidium iodide (PI) and Hoechst 33342 (Dojindo, Kumamoto, Japan) 3 or 7 days after the injection [16]. Endplates were removed with a scalpel blade, and NP tissues were harvested using a biopsy punch. Harvested NP tissues were stained with 4 µM PI and 4 µM Hoechst 33342 for 1 h. PI emits red fluorescence in dead cells, whereas Hoechst 33342 emits blue fluorescence in all cells, regardless of live or dead status. The stained NP tissues were washed with phosphate-buffered saline (PBS) and examined using a confocal laser scanning microscopy system (Nikon A1 and Ti-E, Tokyo, Japan) equipped with a Plan Fluor 20× objective lens (N.A., 0.45; Nikon). The number of dead and all cells in each NP tissue was measured from >3 randomly chosen stacks, each representing a 100-µm projection at a 5-µm interval. ImageJ software (National Institutes of Health, Bethesda, MD, USA) was used for cell number quantification [12], [13]. All the experiments were performed on 6 discs from each treatment group and time point.

#### Histological and TUNEL Assays

Each IVD was prepared for histological and TUNEL assays 7 days after the injection, as described previously [17], [18]. Midsagittal sections (5 µm thick) were stained with hematoxylin and eosin (H&E). In addition, NP cell apoptosis was analyzed by performing TUNEL assays on sagittal paraffin-embedded sections using an In Situ Apoptosis Detection Kit, according to the manufacturer’s recommendations (TaKaRa Bio, Otsu, Japan) [17], [18]. The number of TUNEL-positive cells was counted in 5 independent, randomly selected fields. These values were expressed as the percentage of positive cells relative to the total number of cells [17], [18]. All image assessments were performed by 2 independent blind observers and the quantitative data were presented as the mean of the 3 evaluations (n = 6 discs from each treatment group) [17], [18].

### *In Vivo* study

Under intravenous anesthesia (30 mg/kg) with sodium pentobarbital, rabbit lumbar IVDs were percutaneously punctured. In total, 15 µL [15] of 0.9% saline solution (Otsuka), 1% lidocaine (Astrazeneka), or 0.5% bupivacaine (Astrazeneka) was injected into 2 noncontiguous IVDs (L2/L3 and L4/L5) using a micro syringe with a 26-gauge needle (Hamilton) under fluoroscopic guidance to ensure that the tip of the needle was in the center of IVD [17], [18]. L3/L4 was left intact as a control [17], [18]. A sham procedure was performed by subjecting control animals to IVD needle puncture, but without any reagent. The rabbits were sacrificed 6 or 12 months after the injection.

#### Magnetic Resonance Imaging (MRI) Analysis

Mid-sagittal images of the treated discs were analyzed qualitatively for evidence of degenerative changes using an ultra-high magnetic field strength, 7.0-Tesla MR scanner [Varian Unity Inova (Varian Medical Systems, Palo Alto, CA, USA)], as described previously [17], [18]. The degree of IVD was assessed on sagittal images using the Pfirrmann classification [19]. Quantitative analysis of the sagittal image slices was also processed using the Analyze 10.0 software (AnalyzeDirect, overland Park, KS, USA), as reported previously [17], [18]. The MRI index (product of NP area and average signal intensity) was used to quantify alterations in NP [17], [18]. The quantitative data were expressed as percentage relative to those obtained with untreated control discs [17], [18]. All image assessments were performed by 2 independent blind observers, and the quantitative data were presented as the mean of the 3 evaluations (n = 8 discs from each treatment group/time-point) [17], [18].

#### Histological Analysis

After MRI analysis, each IVD was prepared for histological staining. Midsagittal sections (5 µm thick) were stained with H&E, and with safranin O-fast green [17], [18]. Semiquantitative evaluation of IVD degeneration was performed with a grading system including four categories, with the final grade ranging from 4 (normal) to 12 (highly degenerative) [10]. All image assessments were performed by two independent blind observers, and the quantitative data were presented as the mean of the 3 evaluations (n = 8 discs from each treatment group/time point).

#### Statistical Analysis

All data were expressed as the mean ± standard deviation (SD). Statistical analyses were performed using 1-way analysis of variance, followed by a Tukey–Kramer post-hoc test or the Kruskal–Wallis test for multiple group comparisons. *P*<0.05 was considered statistically significant.

## Results

### Effect of Local Anesthetic Agents on IVD Degeneration in an Organ Culture Model

**Figure 1:**
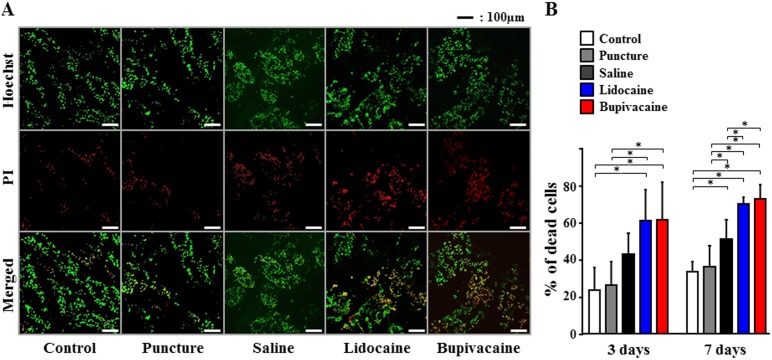
Effects of local anesthetic injection on intervertebral disc (IVD) degeneration in a rabbit IVD organ culture model. Nucleus pulposus (NP) cell death was measured by propidium iodide (PI) (dead cells; red) and Hoechst 33342 (dead and live cells; green) staining and visualized by confocal microscopy at 3 or 7 days after injection. (A) Representative confocal laser scanning micrographs of NP cells after 7 days. (B) The percentage of dead cells. Data are given as means ± SD (*n* = 6). *P<0.05.

We studied the effects of local anesthetic agents routinely used during discoblock on IVD degeneration using a rabbit IVD organ culture model. Saline, 1% lidocaine, or 0.5% bupivacaine were injected into the center of the NP tissue. NP cell death was measured after 3 or 7 days using PI (dead cells; red) and Hoechst 33342 (dead and live cells; green) staining and visualized using confocal microscopy (**Figure 1A**). Quantitative analysis revealed that compared with the untreated control and needle puncture, saline and local anesthetic agents increased the percentage of dead NP cells at 3 and 7 days after the injection. Both anesthetic agents exhibited time-dependent cytotoxicity and compared with the saline solution, significant effects were detected within 7 days (*P*<0.05). After 7 days, the percentage of dead cells increased to 34% with the control, while using lidocaine and bupivacaine increased the percentages of dead cells to 72% and 76%, respectively (Figure 1B).

**Figure 2:**
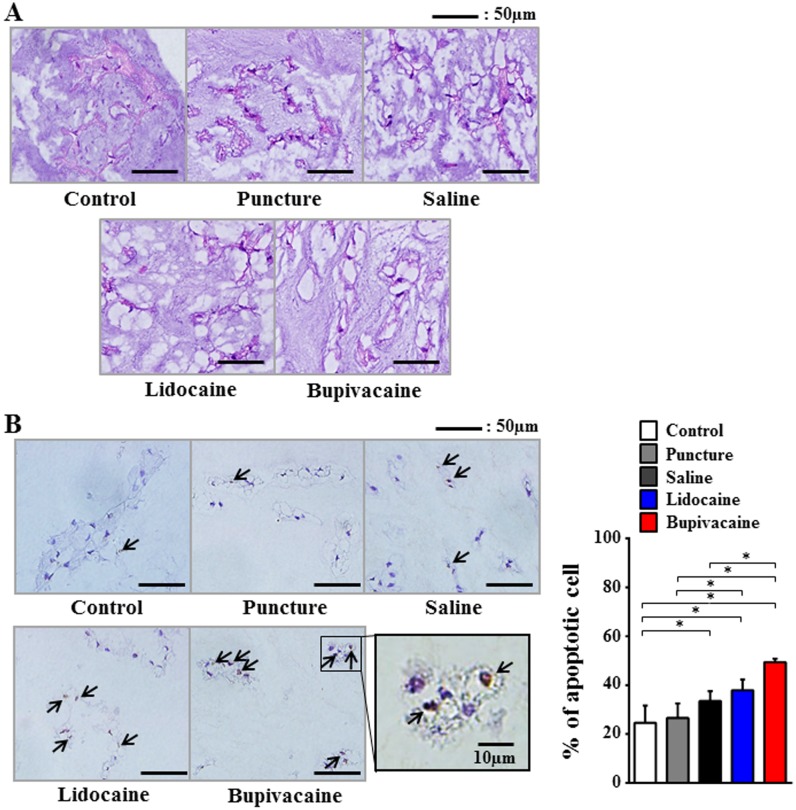
Histological evaluation of intervertebral discs and TUNEL staining of apoptotic nucleus pulposus (NP) cells at 7 days after injection. (A) Hematoxylin and eosin (H&E) staining of NP. (B) TUNEL staining of apoptotic NP cells (arrows). Data are given as means ± SD (*n* = 6). *P<0.05.

Histological analyses were performed 7 days after the injection. H&E staining revealed vacuolization in NP cells and a decrease of the normal gelatinous appearance of the extracellular matrix in the saline and local anesthetic agent groups (**Figure 2A**). TUNEL assay for the detection of NP cell apoptosis revealed that compared with the untreated control and the puncture-only groups, the percentage of TUNEL-positive NP cells significantly increased in the saline, lidocaine, and bupivacaine groups (*P*<0.05). Furthermore, compared with the saline group, the percentage of TUNEL-positive NP cells was significantly higher in the bupivacaine group (*P*<0.05; **Figure 2B**).

### Long-term *In Vivo* Effects of Single Local Injection of Local Anesthetic Agents on IVD Degeneration

**Figure 3:**
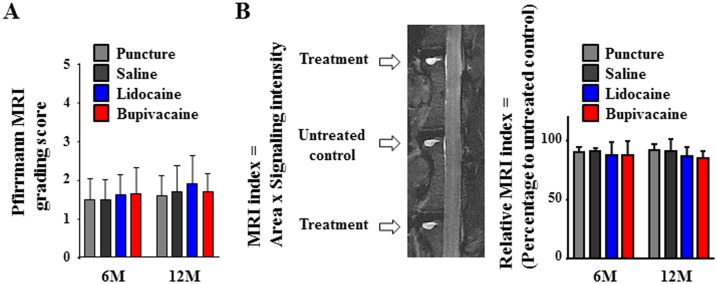
Magnetic resonance imaging (MRI) data analysis of intervertebral disc (IVD)s at 6 or 12 months after injection. (A) Pfirrmann grading score of IVD degeneration. (B) MRI index (product of nucleus pulposus area and average signal intensity) of nucleus pulposus alterations. The numerical values are expressed as percentage compared to untreated control discs (*n* = 8).

We injected local anesthetic agents into the center of rabbit lumbar IVDs under fluoroscopy. Evidence of degenerative changes in rabbit lumbar IVDs were qualitatively analyzed using MRI capturing T2-weighted, mid-sagittal images. The Pfirrmann grading scores [19] time-dependently increased in the saline and both anesthetic agents. However, there was no significant difference among the puncture, saline, and both anesthetic agent groups at 6 or 12 months (**Figure 3A**). The MRI index provides quantitative analysis of the mid-sagittal image [17], [18]. Although values were lower for both the anesthetic agents than for the puncture and saline groups at 6 and 12 months, no significant differences were observed (**Figure 3B**).

**Figure 4:**
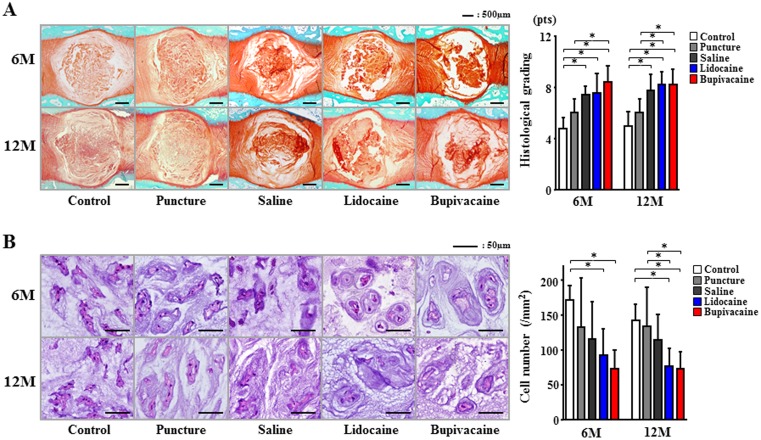
Histological evaluation of intervertebral discs at 6 or 12 months after injection. (A) Safranin O/fast green staining was used for semiquantitative histological scoring. (B) Hematoxylin and eosin (H&E) staining of nucleus pulposus (NP) was used to count the number of NP cells. Data are given as means ± SD (*n* = 8). *P<0.05.

Histological assessment of IVD midsagittal sections revealed degenerative changes in the saline and local anesthetic agent groups, in which 6 months after the injection, many NP contents were lost and the anulus fibrosus had become disorganized. After 12 months, the puncture-only group did not show progression of the degenerative changes, whereas the saline- and local anesthetic-injected groups did. Compared with the untreated control and puncture-only groups, histological scores were significantly increased in the local anesthetic-injected groups (*P*<0.05; **Figure 4A**). However, there was no significant difference between the saline and anesthetic agent groups. H&E staining showed a decrease in the number of NP cells in the puncture, saline, and anesthetic agent groups compared with the untreated control group after 6 months. Compared with the untreated control, a significant decrease was observed in the lidocaine and bupivacaine groups (*P*<0.05). After 12 months, the lidocaine and bupivacaine groups exhibited a significant decrease in the number of NP cells compared with the untreated control and puncture-only groups (*P*<0.05). The number of cells was the lowest in the bupivacaine group; however, there was no significant difference between the saline and bupivacaine groups (**Figure 4B**).

## Discussion

Although discography and discoblock are representative procedures used to diagnose or treat discogenic LBP, these methods were not highly predictive for identifying *bona fide* isolated intradiscal lesions that primarily caused chronic serious LBP [20], [21]. In addition, the potential toxic effects of the agents used during these procedures on human IVDs are not completely understood [6]. Concerns have been raised regarding the safety of these injectates and their possible long-term consequences [22]. Recently, we showed that iotrolan did not exhibit any cytotoxic effect on human healthy NP cells, suggesting that iotrolan should be the radiocontrast agent of choice for safe and effective diagnosis of IVD disorders [6]. In contrast, clinically relevant doses of local anesthetics, such as lidocaine and bupivacaine, induced time- and dose-dependent decreases in viability and increases in the number of apoptotic cells among healthy human NP cells grown in 3-dimensional cultures [6]. Moreover, some *in vitro* studies have reported the cytotoxicity of bupivacaine in IVD cells extracted from patients with IVD disorders [4], [5]. Although lidocaine and bupivacaine are common anesthetic agents used worldwide for the control of LBP in interventional techniques, these observations indicate that local anesthetic agents used to control pain may negatively impact human IVD cells. Thus, further studies of the effects of these agents using organ culture and animal models are warranted to predict what occurs *in vivo* [7].

Wang et al. [23] reported that exposure of mouse IVDs to bupivacaine dramatically decreased cell viability and matrix protein synthesis. Although they used an organotypic culture system that approximated the *in vivo* matrix architecture, the *ex vivo* model did not mimic the discoblock procedure because bupivacaine was not injected using a needle; rather, it was only used as a reagent for IVDs as an alternative to a culture medium in a plate. Here, we used an organ culture model with a clinically relevant procedure; the local anesthetic groups exhibited a time-dependent cytotoxicity and compared with the saline solution, significant effects were detected within 7 days.

In this *ex vivo* model, however, the saline group also exhibited mild deleterious effects on IVDs. Because animal studies confirmed that needle puncture and liquid pressure cause IVD degeneration [10], [11], we used a small needle to reduce the effects of needle puncture on IVD degeneration [11], [24]. However, the pressure applied by the aqueous fluid increased the percentage of dead cells in the organotypic culture model. Therefore, we further constructed *in vivo* animal models to confirm the long-term effects of pressure on IVD degeneration as well as the deleterious effects of local anesthetics. As Carragee et al. [9] performed a longitudinal clinical study to assess the long-term effects of discography up to 10 years, we followed rabbits up to 12 months, which was comparable with 7 to 10 years of the human lifetime [25]. In this study, MRI analysis did not detect any significant differences, while histological analysis revealed that the saline and the local anesthetics promoted degenerative changes. However, there were no significant differences between the saline-injected group and the local anesthetic groups. Therefore, there was no strong evidence that the local anesthetics caused IVD degeneration *in vivo* other than the initial mechanical damage of the pressurized injection. The number of NP cells was the lowest in the bupivacaine group; however, this outcome may be due to damage or a combination of damage and local anesthetics.

Apoptosis plays a central role in the homeostasis of all tissues during normal development and tissue turnover. Recently, we reported that both lidocaine and bupivacaine induced apoptotic cell death in 3-dimensional NP cell–alginate bead composites [6]. Grishko et al. [26] also reported that lidocaine, bupivacaine, and ropivacaine caused apoptosis in cultured human chondrocytes. In this study, we could not perform quantitative analysis of NP cell apoptosis by TUNEL staining *in vivo* because the number of NP cells in local anesthetic-injected IVDs severely decreased (data not shown). However, our *ex vivo* model clearly revealed that bupivacaine significantly induced apoptotic NP cell death, which supported our *in vitro* data [6]. Although the present study did not consider the dose-dependent effects of local anesthetics and did not reveal the *in vivo* deteriorative effects of local anesthetics compared with saline, our recent *in vitro* study demonstrated a dose-dependent increase in the number of apoptotic cells in NP cell–alginate 3D composites exposed to lidocaine or bupivacaine [6]. Because apoptosis plays an important role in the reduction of disc cell number during aging and IVD degeneration [17], [27], the anesthetic agents used for discoblock should be carefully selected for low toxicity toward NP cells in order to minimize tissue degeneration [6].

There were two limitations for this study. First, local anesthetics were introduced into healthy IVDs. It has been shown that cell viability after exposure to bupivacaine is greater in the intact bovine articular cartilage than in chondrocytes suspended in alginate, suggesting that the natural native matrix structure may provide some protection from local anesthetic exposure [28]. NP cells from degenerated human IVD cells may be more susceptible to anesthetic agents than those from healthy IVDs [6]. Second, we injected the same amount of saline or anesthetics into IVDs, but we did not measure the pressure during these injections. We did not find any significant differences in the NP cell numbers among IVDs at different levels (data not shown). However, because not all IVDs are the same size, the pressure alterations after applying the same amount of saline or anesthetics may have differed.

In summary, this is the first *ex vivo* and *in vivo* study to investigate the effects of a single intradiscal injection of lidocaine or bupivacaine on IVD degeneration. Both lidocaine and bupivacaine induced apoptotic NP cell death in the organotypic culture model. However, in the *in vivo* model using healthy IVDs, there was not strong evidence to suggest that discoblock with local anesthetics has the potential of inducing IVD degeneration other than the initial mechanical damage of the pressurized injection. Further studies should be performed to investigate the deteriorative effects of local injection of analgesic agents in degenerated IVDs.
